# Central SELENOT deficiency impairs gonadotrope axis function, sexual behavior, and fertility in male and female mice

**DOI:** 10.1172/jci.insight.189775

**Published:** 2025-11-06

**Authors:** Ben Yamine Mallouki, Loubna Boukhzar, Ludovic Dumont, Azénor Abgrall, Marjorie Gras, Agathe Prieur, David Alexandre, David Godefroy, Yves Tillet, Nathalie Rives, Luca Grumolato, Fatiha Chigr, Youssef Anouar

**Affiliations:** 1 Université Rouen Normandie, Inserm, U1239, Neuroendocrine, Endocrine and Germinal Differentiation and Communication Laboratory, F76000, Rouen, France.; 2 Institute for Research and Innovation in Biomedicine, F76000, Rouen, France.; 3 CNRS UMR 7247, IFCE, INRAE, Université de Tours, Physiologie de la Reproduction et des Comportements, F-37380 Nouzilly, France.; 4 Bioengineering Laboratory, Faculty of Sciences and Techniques, Sultan Moulay Slimane University, Beni Mellal, Morocco.

**Keywords:** Endocrinology, Reproductive biology, Behavior, Fertility, Neuroendocrine regulation

## Abstract

Reproductive disorders can result from a defective action of the neuropeptide gonadotropin-releasing hormone (GnRH), the master regulator of reproduction. We have previously shown that selenoprotein T (SELENOT), a newly described thioredoxin-like selenoprotein highly expressed in endocrine and neuroendocrine cells, plays a role in hormone secretion and neuroprotection. However, whether SELENOT is involved in neuroendocrine regulation in vivo is totally unknown. We found that SELENOT deficiency in the brain impaired sexual behavior, leading to a decline in fertility in both male and female mice. Biochemical and histological analyses of the gonadotrope axis of these mice revealed a higher expression of GnRH, which is associated with circulating luteinizing hormone (LH) excess, and elevated steroid hormones in males and a polycystic ovary syndrome–like phenotype in females. In addition, SELENOT deficiency impaired LH pulse secretion in both male and female mice. These changes were reverted after administration of a GnRH antagonist. Together, our data demonstrate for the first time to our knowledge the role of a selenoprotein in the central control of sexual behavior and reproduction, and identify a redox effector of GnRH neuron activity impacting both male and female reproductive function.

## Abstract 2

Selenoproteins constitute a powerful antioxidant arsenal in mammals. Here, we found that disruption of SELENOT in the brain, a selenoprotein involved in the protection against cellular stress, leads to impairement of sexual behavior and a significant decline in fertility, indicating that selenium and selenoprotein are essential for the control of reproduction by the brain.

## Introduction

Fertility and reproduction are vital physiological functions that ensure species maintenance. In mammals, these functions are under the control of the gonadotrope axis, whose master regulator is the neurohormone gonadotropin-releasing hormone (GnRH) produced in the preoptic area of the hypothalamus (1, 2). This neuropeptide stimulates gonadotropic cells in the anterior pituitary to release the gonadotropin hormones luteinizing hormone (LH) and follicle-stimulating hormone (FSH), the principal hormones that regulate the activity of the gonads. GnRH-producing neurons themselves receive inputs from various brain regions to control reproduction. Indeed, the function of the GnRH network is tightly regulated through the action of different neurotransmitters and neuropeptides, but also steroid hormones released by the gonads, which collectively control the ability to procreate (3, 4). Although recent discoveries have provided new insights into the neuronal pathways involved in the central control of the reproductive function (5, 6), the underlying molecular mechanisms that fine-tune GnRH secretion are only partially elucidated. Further understanding these mechanisms could shed light on the pathophysiological underpinnings of reproductive disorders.

As neuroendocrine secretory cells, hypophysiotropic GnRH neurons and their central regulating systems such as the KNDy neurons (7) are highly active to enable the production of appropriate amounts of GnRH necessary for the permanent control of the gonadotrope axis. In addition, GnRH is secreted in a pulsatile manner, which requires a tight regulation of cell secretory mechanisms (7). As the initial site of hormone and neurohormone synthesis and maturation, the endoplasmic reticulum (ER) plays a pivotal role in neuroendocrine cells. A particular class of ER proteins, named selenoproteins, which incorporates the essential and antioxidant micronutrient selenium, has recently emerged as a central regulator of ER homeostasis and hormone production (8). In particular, we have previously shown that selenoprotein T (SELENOT), a member of ER-resident selenoproteins, is involved in hormone production from pancreatic β (9) and corticotropic cells (10). SELENOT is a novel thioredoxin-like enzyme that is upregulated during neuroendocrine cell differentiation (11, 12). It is widely expressed during development (11) and its genetic ablation in mice results in embryonic lethality before embryonic day 8 (12). While its expression declines in most tissues after birth, high levels are maintained in endocrine and neuroendocrine cells where SELENOT was shown to prevent ER stress (10), suggesting that this protein could play a role in the production and maturation of hormones and neurohormones with vital functions.

Although SELENOT is involved in the ontogenesis of different brain regions (13), exerts neuroprotective effects (12, 14, 15), and participates in endocrine functions, such as insulin (9) and adrenocorticotropin production (10), its role in neuroendocrine regulatory mechanisms remains largely unexplored. We report in the present study that *Selenot* gene disruption in the brain leads to altered sexual behavior and impaired fertility in both male and female mice. These changes were associated with a multi-level dysfunction of the gonadotrope axis, which triggered a polycystic ovary syndrome–like (PCOS-like) phenotype in females and excess testosterone (T) levels in males and females. Our biochemical, histological, and pharmacological studies revealed that these defects are due to increased GnRH input from the medial preoptic area (mPOA) and the median eminence (ME), which leads to altered LH pulsatile release and impaired sexual behavior and fertility. These data uncovered a hitherto unknown role of an ER selenoprotein in the central control of fertility and reproduction in mammals.

## Results

### SELENOT deficiency in the brain alters fertility and sexual behavior.

**Figure 1:**
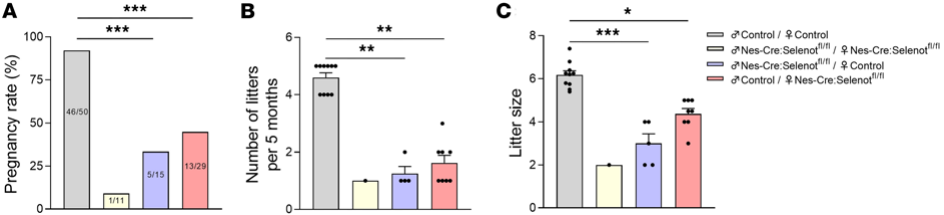
Fertility assessment of brain SELENOT–deficient mice. (**A**) Pregnancy rate corresponding to the percentage of mating resulting in pregnancy. The number of pregnant mice out of the number of mated females is indicated in each bar. Statistical significance for the pregnancy rate was assessed by the χ^2^ test. (**B**) Number of litters for each mated female. (**C**) Litter size. Data are expressed as mean ± SEM and were compared with the nonparametric Kruskal-Wallis test and a posthoc Dunn’s test. *P < 0.05; **P < 0.01; ***P < 0.001. Control females were paired with control males (*n* = 10 per group). *Nes-Cre:Selenot^fl/fl^* females were paired with *Nes-Cre:Selenot^fl/fl^* males (*n* = 8 per group). Control females were paired with *Nes-Cre:Selenot^fl/fl^* males (*n* = 8 per group). *Nes-Cre:Selenot^fl/fl^* females were paired with control males (*n* = 8 per group). Mating was performed during 5 months. Note that no statistical analysis could be performed for the pregnancy rate of the *Nes-Cre:Selenot^fl/fl^* group since only 1 mating out of 11 attempts led to pregnancy in this group, giving birth to 1 litter of 2 pups.

We have previously validated and described a mouse line (*Nes-Cre:Selenot^fl/fl^*) with disruption of the *Selenot* gene in the brain obtained by crossing *Selenot^fl/fl^* mice with mice carrying Cre recombinase under the control of a nestin (*Nes*) promoter (12, 13). In order to investigate the effects of SELENOT inactivation in the brain on mouse fertility, male and female *Nes-Cre:Selenot^fl/fl^* mice were mated during a 5-month period and compared to control littermate animals (control group). As shown in Figure 1A, the pregnancy rate was dramatically reduced in the *Nes-Cre: Selenot^fl/fl^* group (1 out of 11 mated females) compared with the control group, which showed normal reproductive behavior (46 out of 50 females). In continuous mating, the *Nes-Cre:Selenot^fl/fl^* group showed impaired fertility, as indicated by the birth of only 1 litter with 2 pups during the mating period (Figure 1, B and C). We then tested the fertility of male *Nes-Cre:Selenot^fl/fl^* mice after mating with female control mice. As shown in Figure 1A, the number of female control mice becoming pregnant when mated with male *Nes-Cre:Selenot^fl/fl^* mice was significantly lower (5 out of 15 females) compared with those mated with control males. In continuous mating, control females had fewer litters when mated with mutant males as compared with control males, and the number of pups per litter produced over 5 months was also significantly lower (Figure 1, B and C). The fertility of *Nes-Cre:Selenot^fl/fl^* females was also assessed after mating with male control mice. In comparison with control females, the number of *Nes-Cre:Selenot^fl/fl^* females that became pregnant was significantly reduced (13 out of 29 females; Figure 1A). In continuous mating, female *Nes-Cre:Selenot^fl/fl^* mice mated with male control had fewer litters (Figure 1B) and the number of pups per litter produced over 5 months was significantly lower compared with control females (Figure 1C). Together, these data revealed that SELENOT deficiency in the brain alters male and female fertility in mice.

**Figure 2:**
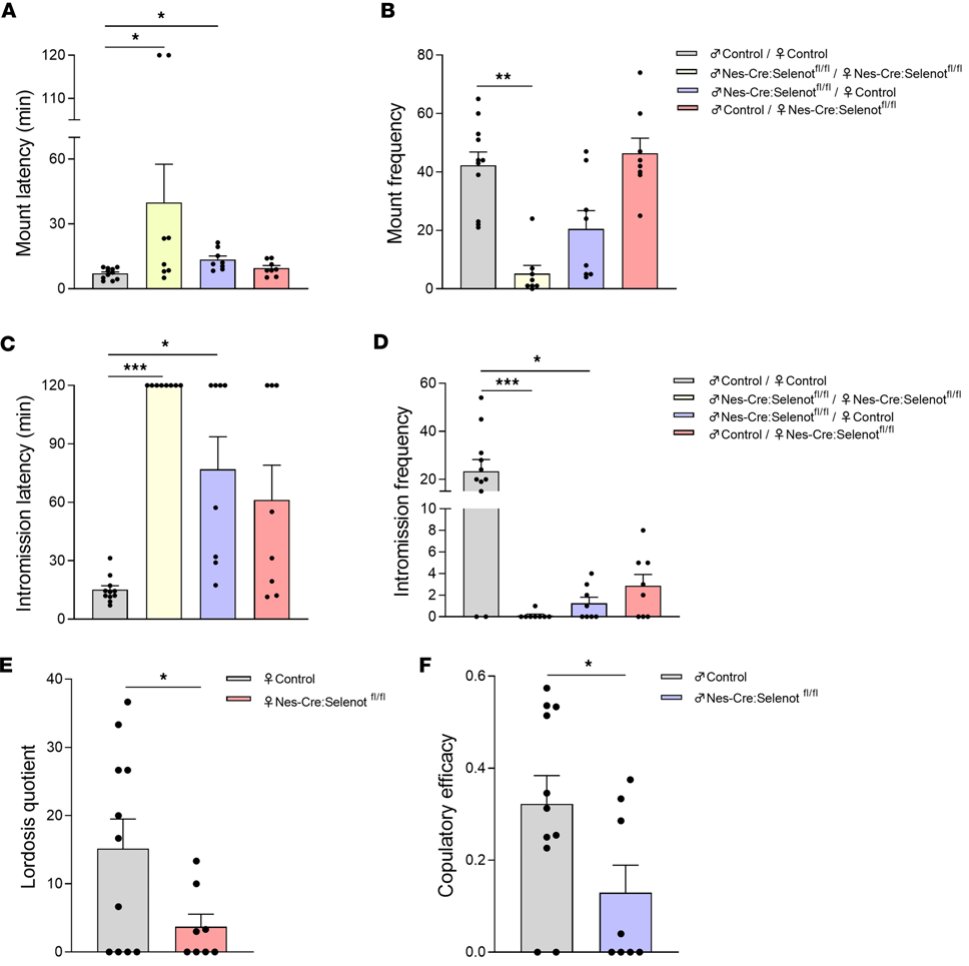
Sexual behavior of brain SELENOT–deficient mice. (**A**) Mount latency is represented for the different groups of mice. (**B**) Mount frequency. (**C**) Intromission latency. (**D**) Intromission frequency. (**E**) The lordosis quotient of female *Nes-Cre:Selenot^fl/fl^* mice was calculated as the lordosis number/number of mounts. (**F**) Copulatory efficacy was calculated as the intromission frequency divided by mount frequency + intromission frequency. For these analyses, male and female *Nes-Cre:Selenot^fl/fl^* mice (*n* = 8 for each group) and male and female control mice (*n* = 11 for each group) were used. The data are expressed as mean ± SEM and were compared using the nonparametric Kruskal-Wallis test with a post hoc Dunn’s test (**A**–**D**) and unpaired, 2-tailed Student’s *t* test (**E** and **F**). *P < 0.05; **P < 0.01; ***P < 0.001.

In line with these data, couples of *Nes-Cre:Selenot^fl/fl^* mice exhibited longer mount latency and lower mount frequency compared with control littermates (Figure 2, A and B). In addition, these mice exhibited increased intromission latency and almost no intromissions compared with the control group (Figure 2, C and D). The behavioral changes observed in *Nes-Cre:Selenot^fl/fl^* mice were due to sexual impairments in both male and female mutant mice. Indeed, *Nes-Cre:Selenot^fl/fl^* males mated with female control mice showed an increase in mount and intromission latencies (Figure 2, A–C), and a decrease in mount and intromission frequencies (Figure 2, B–D). For female *Nes-Cre:Selenot^fl/fl^* mice, these parameters were not significantly modified, as they depend mostly on control males used in mating (Figure 2, A–D). However, the lordosis quotient was significantly decreased in *Nes-Cre:Selenot^fl/fl^* females compared with control females (Figure 2E), indicating an altered receptivity of these females. Also, a significant decrease in the copulatory efficacy was found for *Nes-Cre:Selenot^fl/fl^* males compared with control males (Figure 2F). Thus, central SELENOT is necessary for normal sexual behavior of both male and female mice.

### Nes-Cre:Selenot^fl/fl^ mice exhibit increased GnRH levels.

**Figure 3:**
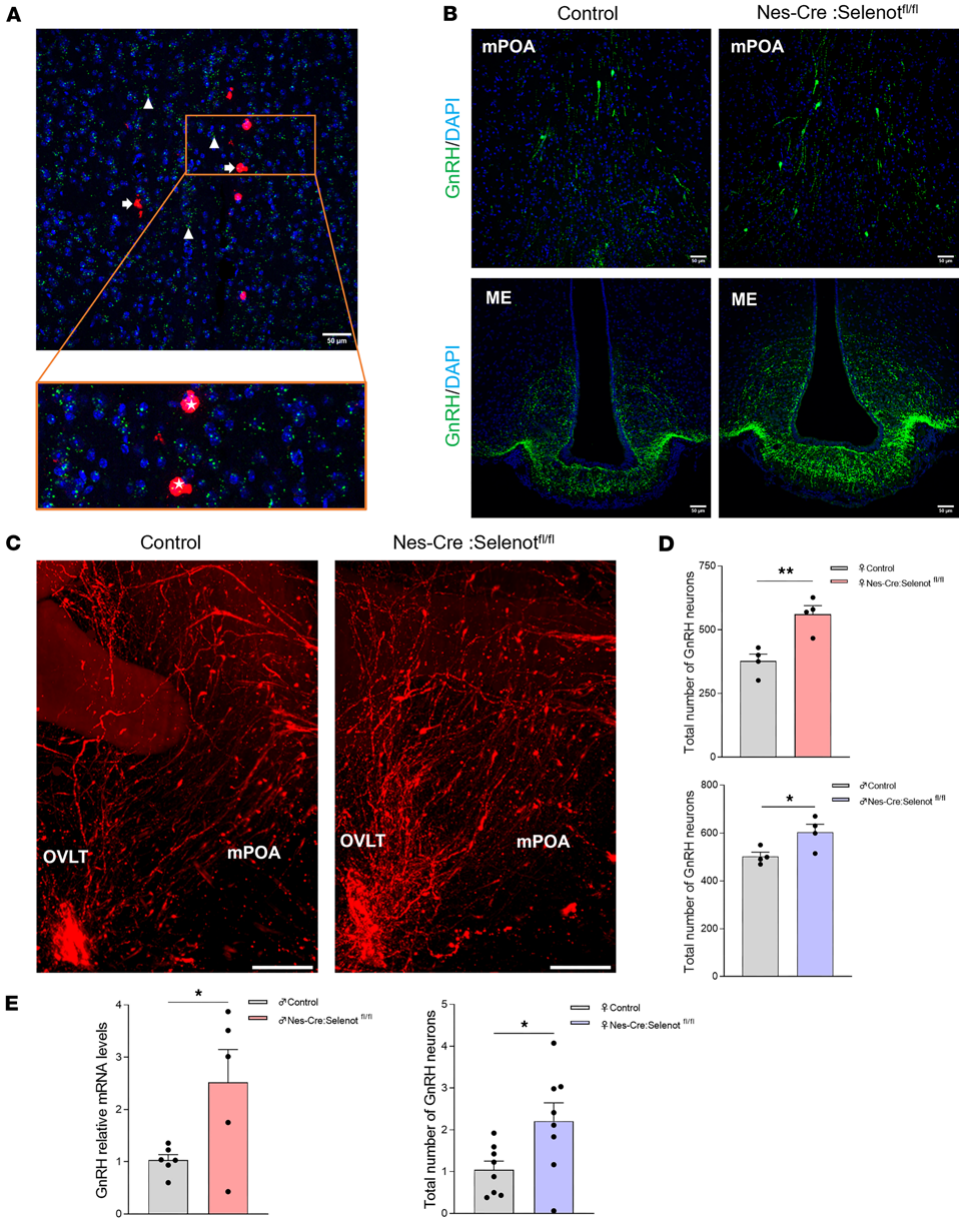
Distribution of GnRH neurons in the hypothalamus of *Selenot*^–/–^ mice. (**A**) RNAScope analysis of SELENOT and GnRH mRNA expression in the mPOA. *Gnrh* mRNA is labeled in red (arrows) and *Selenot* mRNA is in green (arrowheads). Colabeled neurons expressing both mRNAs are indicated in the inset by asterisks. Nuclei are labeled in blue by DAPI. Scale bar: 50 μm. The images below are a higher-magnification view of the box in the top image (×5 of the original image). (**B**) Coronal sections at the mPOA and ME were immunostained for GnRH (green). Nuclei were stained in blue using DAPI. Scale bars: 50 μm. (**C**) 3D sagittal view of GnRH labeling with anatomical references in adult control and *Nes-Cre:Selenot^fl/fl^* mice. Scale bars: 500 μm. OVLT, organum vasculosum of the lamina terminalis; mPOA, median preoptic area. (**D**) Total number of hypothalamic GnRH neurons in adult female and male control and *Nes-Cre:Selenot^fl/fl^* mice (*n* = 4 per group). (**E**) *Gnrh* mRNA levels in the hypothalamus of control and *Nes-Cre:Selenot^fl/fl^* adult female (*n* = 6 and 5 per group, respectively) and male (*n* = 8 per group) mice. Statistical analysis was performed using unpaired, 2-tailed Student’s *t* test. *P < 0.05, **P < 0.01.

We then asked whether GnRH neurons, which control the gonadotrope axis and sexual behavior (1), express SELENOT. RNAScope analysis showed indeed that virtually all hypothalamic GnRH neurons of the mPOA exhibited *Selenot* mRNA expression (Figure 3A). To determine whether disruption of the *Selenot* gene in the brain could affect hypothalamic GnRH neuron distribution and density, we analyzed GnRH immunoreactivity in coronal sections of the hypothalamus from adult *Nes-Cre:Selenot^fl/fl^* and control mice. These studies revealed a higher immunoreactive signal in the mPOA and ME of *Nes-Cre:Selenot^fl/fl^* mice compared with littermate controls (Figure 3B). To confirm these findings, we compared the number of GnRH neurons in the hypothalamus of adult male and female *Nes-Cre:Selenot^fl/fl^* mice with that of control mice using iDISCO-based 3D immunohistochemistry (15). The characteristic rostro-caudal distribution pattern of the GnRH neurons and the arborization of their processes were demonstrated by reconstructing series of optical slices in the hypothalamus of adult *Nes-Cre:Selenot^fl/fl^* and control mice (Figure 3C). This analysis revealed that the number of GnRH neurons was higher in male and female *Nes-Cre:Selenot^fl/fl^* compared with control mice (Figure 3D). This increase in the immunoreactive neuropeptide was accompanied by an increase in the *Gnrh* mRNA levels, as indicated by qPCR analysis of hypothalamic mRNA (Figure 3E), indicating that SELENOT deficiency alters *Gnrh* gene expression.

### Nes-Cre:Selenot^fl/fl^ mice exhibit abnormal LH pulse secretion and altered estrous cyclicity.

**Figure 4:**
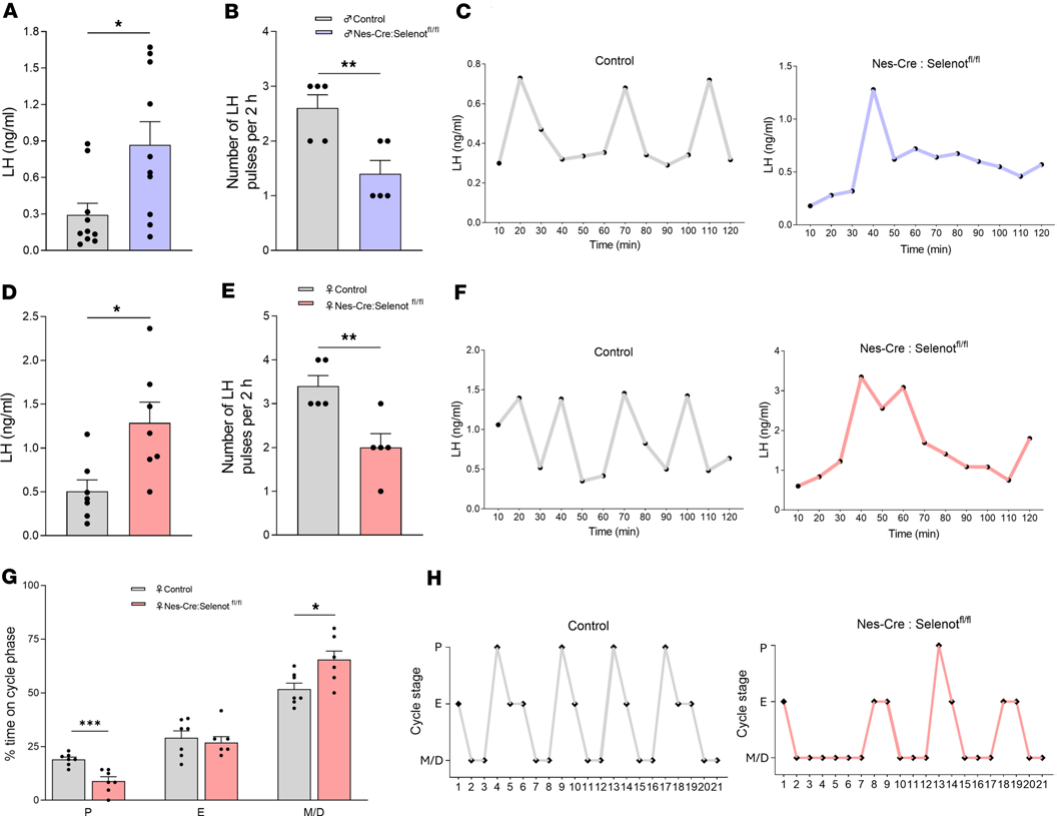
LH circulating levels and estrous cyclicity in brain SELENOT–deficient mice. (**A**) Plasma LH levels in adult males (*n* = 10 per group). (**B**) Number of LH pulses during 2 hours in adult males (*n* = 5 per group). (**C**) Representative graphs of LH pulsatility during a 2-hour interval for male control and *Nes-Cre:Selenot^fl/fl^* mice. (**D**) Plasma LH levels in adult female control and *Nes-Cre:Selenot^fl/fl^* mice (*n* = 7 per group). (**E**) Number of LH pulses during 2 hours in females at diestrus (*n* = 5 per group). (**F**) Representative graphs of LH pulsatility during a 2-hour interval for adult female control and *Nes-Cre:Selenot^fl/fl^* mice. (**G**) Percentage of time spent in each estrous cycle stage (E, estrus; M/D, metestrus/diestrus; P, proestrus) (*n* = 7 per group). (**H**) Representative estrous cyclicity of control and *Nes-Cre:Selenot^fl/fl^* mice during 21 consecutive days. Statistical analysis was performed using unpaired, 2-tailed Student’s *t* test. *P < 0.05, **P < 0.01, ***P < 0.001.

To determine whether the higher intensity of GnRH immunoreactivity observed in the mPOA and ME would translate into a higher LH output, circulating LH levels were measured in male and female *Nes-Cre:Selenot^fl/fl^* mice (Figure 4). In males, LH levels were indeed higher in *Nes-Cre:Selenot^fl/fl^* compared with control mice (Figure 4A). In addition, a significantly lower LH pulse frequency was observed in *Nes-Cre:Selenot^fl/fl^* males compared with control males (Figure 4, B and C). Similarly, LH levels measured at diestrus were markedly elevated in *Nes-Cre:Selenot^fl/fl^* females compared with controls (Figure 4D). *Nes-Cre:Selenot^fl/fl^* females also had a significantly lower LH pulse frequency as compared with controls (Figure 4, E and F). These results indicate that SELENOT deficiency impacts the function of the hypothalamic-hypophyseal system in both male and female mice. Because LH concentration regulates the length and order of the different phases of the estrous cycle in females (16), we wondered whether the changes observed in circulating LH levels would lead to impairment of the reproductive cycle. We found that a large majority of *Nes-Cre:Selenot^fl/fl^* females exhibit severely disrupted estrous cyclicity (oligo-ovulation). *Nes-Cre:Selenot^fl/fl^* females rarely reached the preovulatory stage of the estrous cycle and exhibited prolonged duration of metestrus and diestrus, compared with control females (Figure 4, G and H).

### Nes-Cre:Selenot^fl/fl^ mice exhibit abnormal hormone levels and defective follicular development.

**Figure 5:**
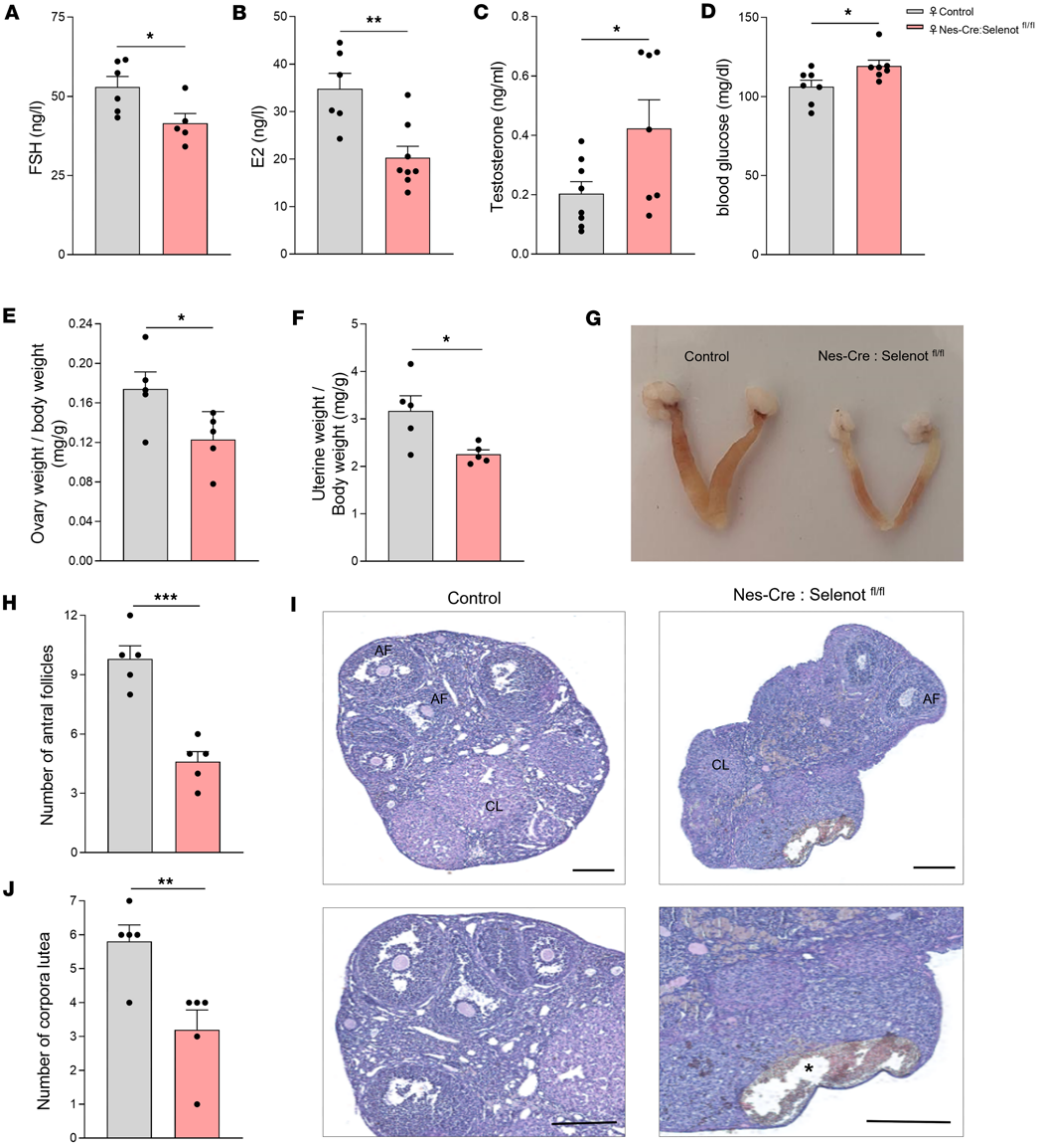
Hormone levels and ovarian morphology of brain SELENOT–deficient mice. (**A**) Plasma FSH levels in adult female control and *Nes-Cre:Selenot^fl/fl^* mice (*n* = 6 and 5, respectively). (**B**) Plasma E_2_ concentration in adult female control and *Nes-Cre:Selenot^fl/fl^* mice (*n* = 6 and 8, respectively). (**C**) Plasma T levels measured in adult female control and *Nes-Cre:Selenot^fl/fl^* mice (*n* = 8 and 7, respectively). (**D**) Blood glucose levels were measured in fasted adult female control and *Nes-Cre:Selenot^fl/fl^* mice (*n* = 7 per group). (**E**) Ratio of ovary weight to body weight in control and *Nes-Cre:Selenot^fl/fl^* mice (*n* = 5 per group). (**F**) Ratio of uterine weight to body weight in control and *Nes-Cre:Selenot^fl/fl^* mice (*n* = 5 per group). (**G**) Gross structure of the ovaries and uteri is shown. (**H**) Quantitative analysis of antral follicle (AF) and corpora lutea (CL) in control and *Nes-Cre:Selenot^fl/fl^* mice (*n* = 5 per group). (**I**) H&E staining of the ovarian tissue morphology of adult control and *Nes-Cre:Selenot^fl/fl^* mice. Scale bars: 200 μm. Ovarian sections containing AF and CL are shown. Asterisks denote ovarian cysts. Data are presented as mean ± SEM. Statistical analysis was performed with unpaired, 2-tailed Student’s *t* test. *P < 0.05, **P < 0.01, ***P < 0.001.

To further characterize the gonadotrope axis in female *Nes-Cre:Selenot^fl/fl^* mice, we measured circulating FSH, estradiol (E_2_), and T levels. While FSH and E_2_ were significantly decreased in *Nes-Cre:Selenot^fl/fl^* compared with control mice (Figure 5, A and B), T concentration was higher in *Nes-Cre:Selenot^fl/fl^* compared with control mice (Figure 5C). These data suggest that the subfertility and altered sexual behavior of female mice with SELENOT inactivation in the brain is due to abnormal hormonal levels in the whole gonadotrope axis.

Because glycemia dysregulation could be also associated with certain reproductive disorders, we measured blood glucose in the knockout animals, and found that glycemia was higher in these mice compared with control littermates (Figure 5D).

To determine whether the abnormal circulating FSH and E_2_ levels observed in the female *Nes-Cre:Selenot^fl/fl^* mice impinge on ovary and uterine morphology, we first assessed the ratios of ovary and uterine weight to body weight, markers of circulating FSH and estrogen concentrations, respectively (17). We found that both markers were affected in *Nes-Cre:Selenot^fl/fl^* mice since they were both significantly reduced compared with control littermates (Figure 5, E–G). Ovarian histology of *Nes-Cre:Selenot^fl/fl^* females showed abnormalities consistent with their anovulatory phenotype, with the presence of fewer antral follicles (containing a single large antrum) and significantly less postovulation corpora lutea compared with control females (Figure 5H). In addition, cysts were observed in the ovaries of brain SELENOT–deficient mice (Figure 5I).

### Male Nes-Cre:Selenot^fl/fl^ mice exhibit abnormal hormone levels and testicular changes.

**Figure 6:**
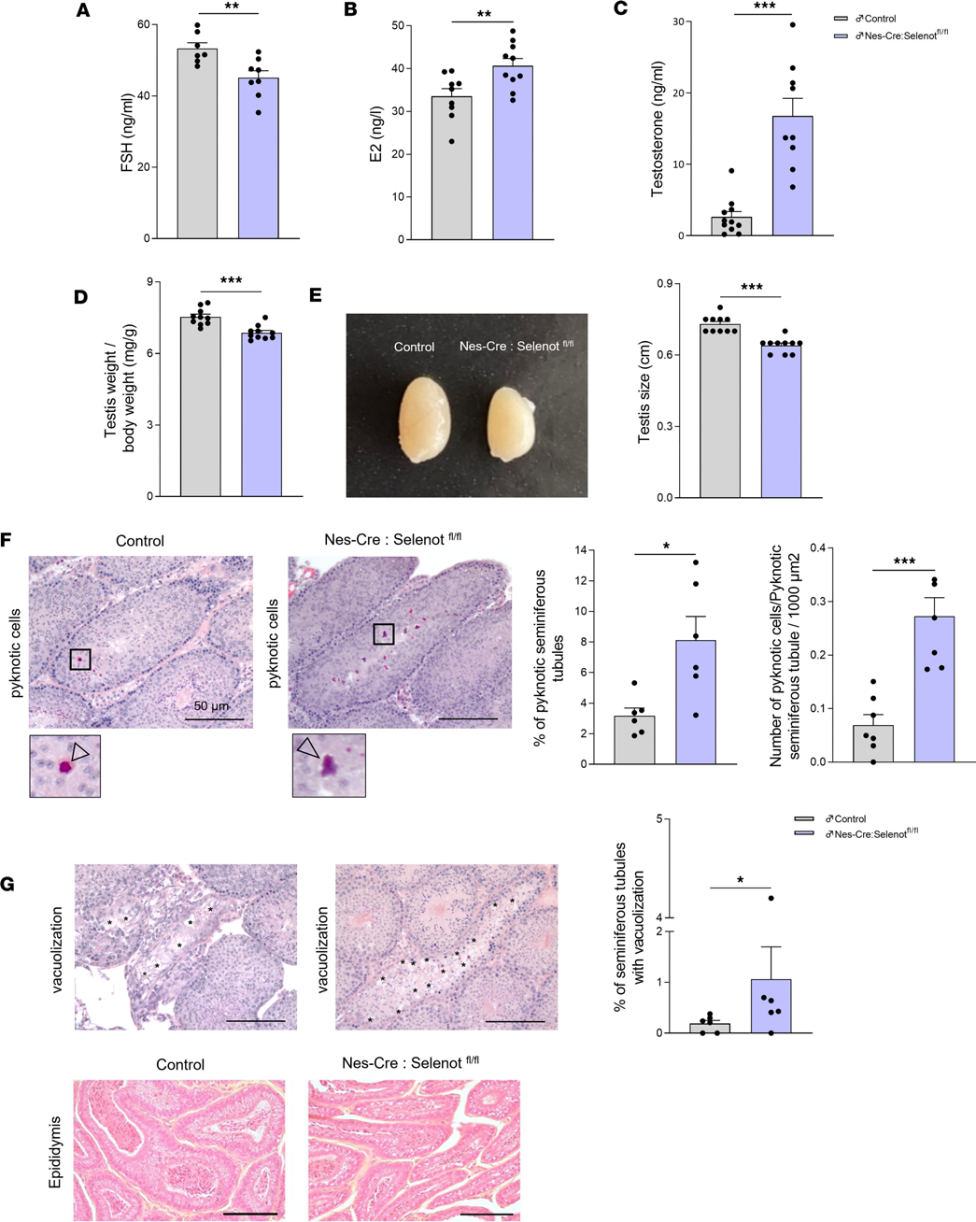
Hormone levels and testis morphology of brain SELENOT–deficient mice. (**A**) Plasma FSH concentration in adult male control and *Nes-Cre:Selenot^fl/fl^* mice (*n* = 7 and 8, respectively). (**B**) Plasma E_2_ concentration in adult male control and *Nes-Cre:Selenot^fl/fl^* mice (*n* = 9 and 10, respectively). (**C**) Plasma T concentration in adult male control and *Nes-Cre:Selenot^fl/fl^* mice (*n* = 11 and 9, respectively). (**D**) Ratio of testis weight to body weight in control and *Nes-Cre:Selenot^fl/fl^* mice (*n* = 10 per group). (**E**) Representative image showing the morphology of the testis and the corresponding measurement of the size in control and *Nes-Cre:Selenot^fl/fl^* mice. (**F**) Histological evaluation using H&E and saffron staining of testicular tissue from adult control and *Nes-Cre:Selenot^fl/fl^* mice. Cell pyknotic nuclei (arrowhead in the inset) were observed in seminiferous tubules of control and *Nes-Cre:Selenot^fl/fl^* mice. The images below are a higher-magnification view of the boxes in the top images (×10 of the original image). Stereological analyses of testicular tissues from control and *Nes-Cre:Selenot^fl/fl^* mice was used to determine the percentage of pyknotic seminiferous tubules, the number of pyknotic cells/1000 μm^2^ for seminiferous tubules containing at a minimum 1 pyknotic cell. (**G**) Vacuolizations in seminiferous tubules (asterisks) were analyzed in testicular tissues from control and *Nes-Cre:Selenot^fl/fl^* mice, and the percentage of seminiferous tubules with vacuolization was determined. (**H**) H&E staining of cauda epididymides sections from adult male control and *Nes-Cre:Selenot^fl/fl^* mice. Scale bars: 50 μm. The values are expressed as the mean percentage ± SEM, with *n* = 6 and 5, respectively, per group (for each animal, 4 testicular tissue pieces and 2 slices/tissue piece were analyzed). Statistical analysis was performed with unpaired, 2-tailed Student’s *t* test. *P < 0.05, **P < 0.01, ***P < 0.001.

Because male *Nes-Cre:Selenot^fl/fl^* mice also displayed impaired sexual behavior and fertility, and higher levels of GnRH and LH compared with control, we investigated the male gonadotrope axis activity and testis morphology. The data showed that, as in females, FSH concentration was lower in *Nes-Cre:Selenot^fl/fl^* compared with control males (Figure 6A). In addition, E_2_ levels were higher and T levels were markedly elevated in *Nes-Cre:Selenot^fl/fl^* males compared with control males (Figure 6, B and C). These results showed that, as in females, SELENOT deficiency in the male brain alters the whole activity of the gonadotrope axis, which impacts male fertility as described above.

At the level of the gonads, the weight and size of the testis were significantly reduced in *Nes-Cre:Selenot^fl/fl^* males compared with control littermates (Figure 6, D and E). Histologic examinations were carried out on H&E- and saffron-stained sections of testis (Figure 6F). The overall histology of the testicular tissue in *Nes-Cre:Selenot^fl/fl^* mice did not seem to be altered (Figure 6F). However, a higher percentage of pyknotic seminiferous tubules was observed in *Nes-Cre:Selenot^fl/fl^* testicular tissue compared with control (Figure 6F). The number of pyknotic cells per area of seminiferous tubules containing at a minimum 1 pyknotic cell was also higher in *Nes-Cre:Selenot^fl/fl^* testes (Figure 6F). In addition, an important vacuolization was observed in seminiferous tubules within the testicular tissue of *Nes-Cre:Selenot^fl/fl^* mice (Figure 6G). No apparent difference in epididymal anatomy was seen between *Nes-Cre:Selenot^fl/fl^* and control animals (Figure 6H).

### GnRH antagonist treatment restores the neuroendocrine phenotype and ovarian morphology of Nes-Cre:Selenot^fl/fl^ mice.

**Figure 7:**
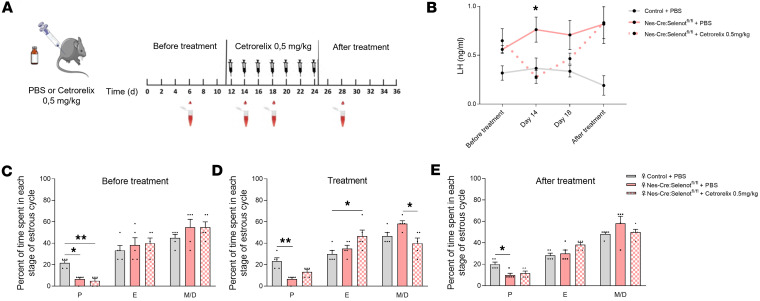
Treatment with a GnRH antagonist restored the neuroendocrine phenotype of female brain SELENOT–deficient mice. (**A**) Schematic of the experimental design showing the 3 steps of the protocol, before, during, and after i.p. injection of 0.5 mg/kg of cetrorelix acetate or PBS. Female *Nes-Cre:Selenot^fl/fl^* mice were injected i.p. for 12 days with cetrorelix acetate (every other day). Tail blood samples were collected for measurement of LH, once before the start of the treatment and then on days 2 and 6 of treatment, as well as on day 4 after the last injection (*n* = 5 per group). (**B**) Time course of serum LH concentration in *Nes-Cre:Selenot^fl/fl^* mice before the beginning of the treatment, 2 and 6 days after the first injection of cetrorelix, and after discontinuation of the drug antagonist. Blood from control and *Nes-Cre:Selenot^fl/fl^* mice that received PBS injection as a control was collected during the same temporal windows as for cetrorelix treatment. (**C**–**E**) Percentage of time spent in each estrous cycle was determined for each group of animals and in each condition (E, estrus; M/D, metestrus/diestrus; P, proestrus). Statistical analysis was performed using the nonparametric Kruskal-Wallis test with a post hoc Dunn’s test. *P < 0.05; **P < 0.01.

Since our results uncovered a hyperactivation of GnRH neurons in *Nes-Cre:Selenot^fl/fl^* mice, we reasoned that competing with endogenous GnRH for binding to membrane receptors on gonadotropic cells could decrease the rate of LH release in female mutant mice, thus restoring physiological LH levels and estrous cyclicity in these animals. To test this hypothesis, we monitored LH levels and estrous cyclicity in *Nes-Cre:Selenot^fl/fl^* females for 12 days before, during, and after the treatment with the GnRH antagonist cetrorelix. The mice received cetrorelix acetate at 0.5 mg/kg, i.p. every second day (Figure 7A). The 12-day periods of treatment with the vehicle or cetrorelix ensured the observation of 3 consecutive estrous cycles at each period, and such antagonist treatment has been previously shown to normalize LH release and estrous cyclicity (18). Tail-blood samples were collected for LH measurements in brain *Nes-Cre:Selenot^fl/fl^* and control females twice during the GnRH antagonist treatment, and once before and after the treatment period (Figure 7A). Following intermittent GnRH antagonist treatment (0.5 mg/kg), the high mean LH values initially observed in female *Nes-Cre:Selenot^fl/fl^* mice were significantly reduced to reach levels indistinguishable from those of control (Figure 7B). When the treatment was discontinued, LH levels increased again to levels similar to those observed before the treatment (Figure 7B).

As described above, *Nes-Cre:Selenot^fl/fl^* females spent a shorter time in proestrus as compared with control females (Figure 7C). When *Nes-Cre:Selenot^fl/fl^* females were injected with 0.5 mg/kg of GnRH antagonist, almost normal estrous cyclicity was recovered with a proestrus and metestrus/diestrus times comparable to those of control animals (Figure 7D). When the antagonist treatment was stopped, proestrus and estrous times were altered (Figure 7E).

### GnRH antagonist treatment normalizes LH pulsatility in both male and female Nes-Cre:Selenot^fl/fl^ mice.

**Figure 8:**
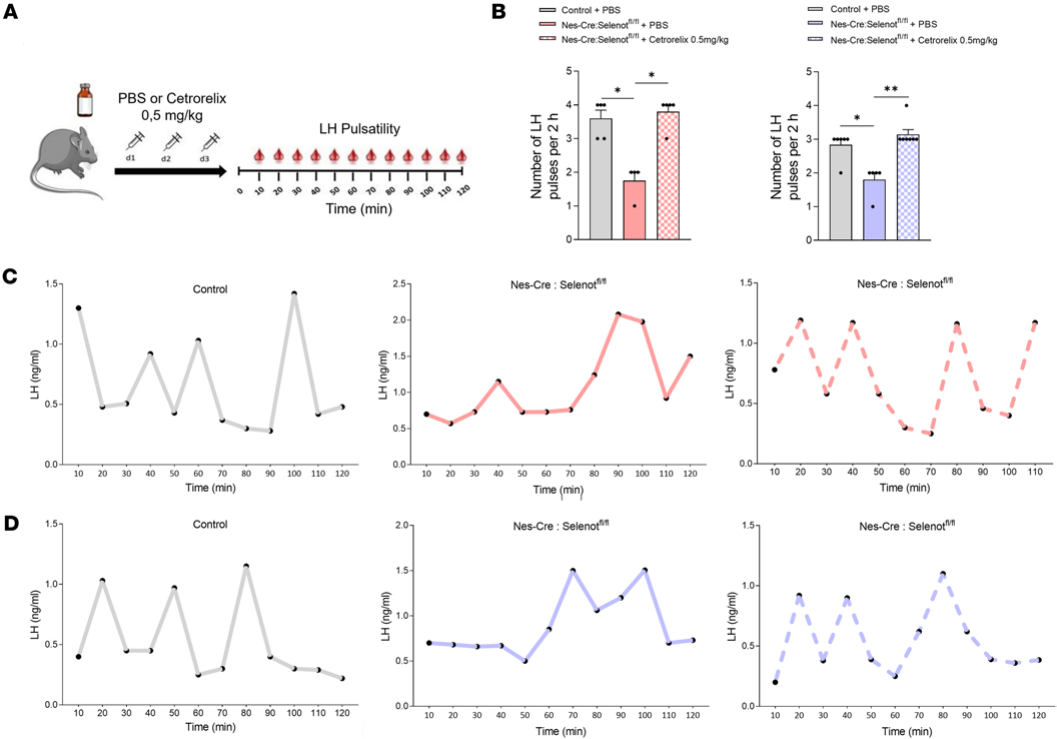
Treatment of male and female brain SELENOT–deficient mice with a GnRH antagonist restored LH pulsatility. (**A**) Schematic of the experimental design. Female and male *Nes-Cre:Selenot^fl/fl^* mice were injected i.p. with cetrorelix acetate or PBS every day during 3 days. Tail blood samples were collected after the treatment, every 10 minutes during 2 hours for measurement of LH concentration. (**B**) Representative graphs of LH pulsatility in female control and *Nes-Cre:Selenot^fl/fl^* mice. (**C**) Representative graphs of LH pulsatility in male control and *Nes-Cre:Selenot^fl/fl^* mice. (**D**) Number of LH pulses in adult females at diestrous (*n* = 5 control + PBS, 4 *Nes-Cre:Selenot^fl/fl^* + PBS, and 5 *Nes-Cre:Selenot^fl/fl^* + cetrorelix acetate) and males (*n* = 6/5/7) during 2 hours. Statistical analysis was performed using the nonparametric Kruskal-Wallis test with a post hoc Dunn’s test. *P < 0.05; **P < 0.01.

To assess whether the reduced LH pulse frequency observed in male and female *Nes-Cre:Selenot^fl/fl^* mice is due to increased GnRH activity, we administered the GnRH antagonist cetrorelix acetate at 0.5 mg/kg. i.p. every day for 3 days, and measured LH pulsatility from blood samples (Figure 8A). As shown in Figure 8B, cetrorelix acetate restored the LH pulse frequency in both female and male *Nes-Cre:Selenot^fl/fl^* mice as compared with control littermates. Indeed, female and male *Nes-Cre:Selenot^fl/fl^* mice treated with the antagonist displayed a similar number of LH pulses compared to control mice (Figure 8, C and D). These results indicate that the altered LH pulse secretion observed in male and female brain SELENOT-deficient mice could be ascribed to the impaired GnRH activity.

### Kisspeptin gene expression is altered in the anteroventral paraventricular nucleus but not the arcuate nucleus in male and female Nes-Cre:Selenot^fl/fl^ mice.

**Figure 9:**
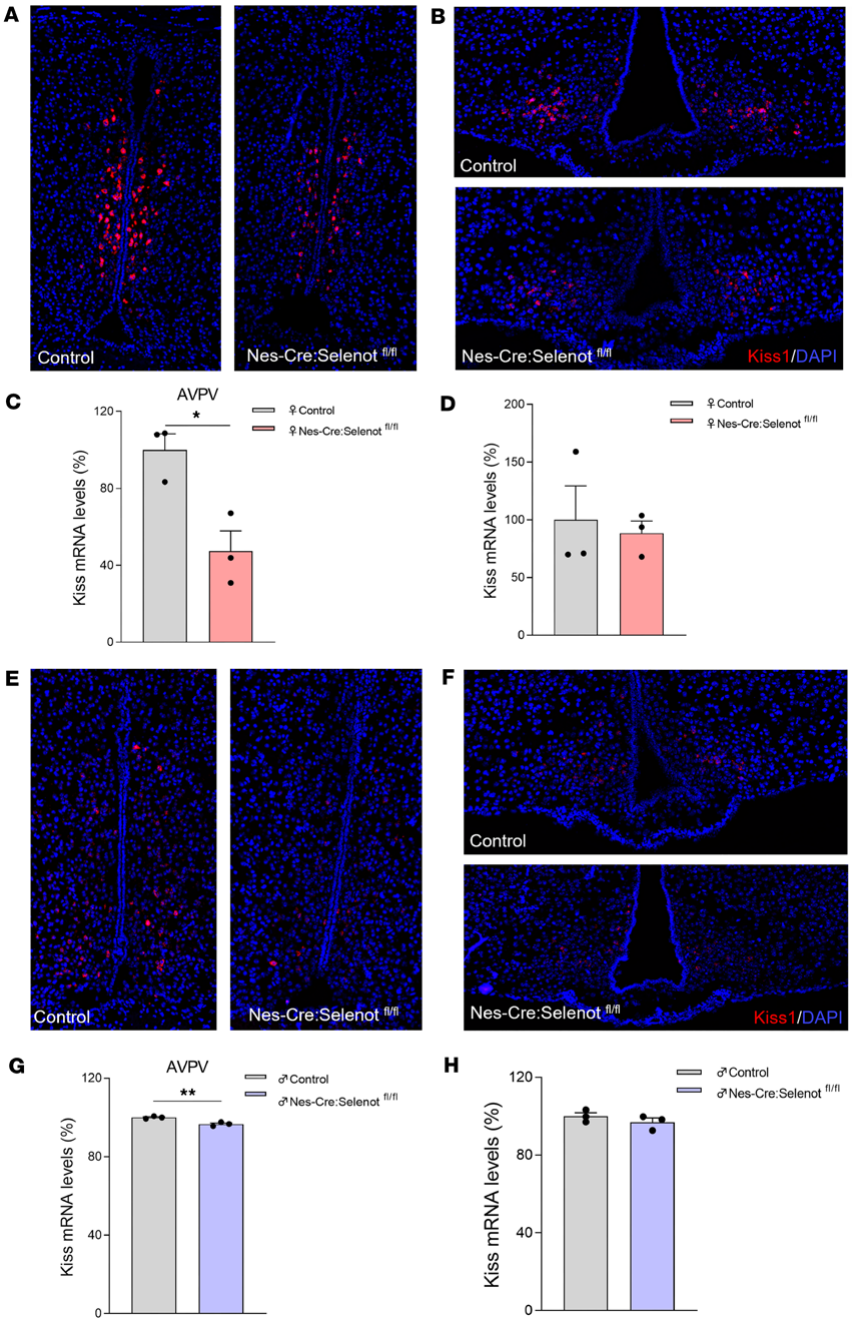
Analysis of the expression of kisspeptin mRNA by RNAScope in situ hybridization. (**A**–**D**) RNAScope analysis of kisspeptin mRNA expression in the AVPV and ARC from adult female control and *Nes-Cre:Selenot^fl/fl^* mice (*n* = 3). (**E**–**H**) RNAScope analysis of kisspeptin mRNA expression in the AVPV and ARC from adult male control and *Nes-Cre:Selenot^fl/fl^* mice (*n* = 3). Kisspeptin mRNA is labeled in red and nuclei are labeled in blue by DAPI. Original magnification, ×20. Statistical analysis was performed using an unpaired, 2-tailed Student’s *t* test. *P < 0.05, **P < 0.01.

To further elucidate the mechanisms involved in the changes in GnRH neuron activity, we examined the hypothalamic anteroventral paraventricular nucleus (AVPV) and arcuate nucleus (ARC) expression of the gene encoding kisspeptin, which is known to regulate LH surge and pulsatility, respectively, through GnRH regulation. At diestrus, kisspeptin mRNA levels were significantly reduced in the AVPV of the mutant mice compared with controls in both females (Figure 9, A and C) and males (Figure 9, E and G), while there was no significant variation in these mRNAs in the ARC, neither in female (Figure 9, B and D) nor in male mutant mice (Figure 9, F and H).

## Discussion

In this report, we provide the first evidence to our knowledge for the neuroendocrine role of a selenoprotein, i.e., SELENOT, in the control of reproduction using male and female transgenic animals with *Selenot* gene disruption in the brain. SELENOT is an essential thioredoxin-like enzyme initially identified in silico (19) and then as a PACAP-stimulated gene during neuroendocrine cell differentiation (11). SELENOT, whose global gene knockout is embryonic lethal (12), has been shown to play a prominent role in the brain in dopaminergic neuron distribution and survival (12, 15), and in the periphery in hormone secretion (9–11). However, the role of central *Selenot* gene expression in vital functions involving neuroendocrine circuits has not been explored yet. In fact, the role of selenoproteins in neuroendocrine mechanisms remains largely unknown, although selenium levels and certain selenoproteins have been shown to influence several critical physiological functions in the periphery, including reproduction (20–22).

Although brain-specific *Selenot*-knockout mice are viable, in contrast with the total knockout mice which die in utero, these animals failed to reproduce normally. This intriguing result prompted us to investigate the gonadotrope axis function of male and female knockout mice. Because GnRH is the master regulator of this axis, we examined its expression and distribution pattern in SELENOT-deficient and control mice using immunohistochemistry and 3D light-sheet imaging after transparisation of cerebral tissue. Analysis of the signals and quantification of 3D images revealed a higher GnRH immunoreactivity in the mPOA and their fibers in the ME in *Selenot*-knockout mice compared with control littermates in both sexes. This result was confirmed by quantitative PCR, which showed higher expression of the GnRH gene in the hypothalamus of the mutant mice. These findings suggested that SELENOT plays a major role in the central control of reproduction.

Because the higher GnRH levels of *Nes-Cre:Selenot^fl/fl^* compared with control mice could impact the gonadotrope axis at the pituitary and gonadal levels, we analyzed the circulating hormones produced by these tissues. LH levels, which are tightly correlated to those of GnRH, were also significantly increased, with changes in LH pulsatility in both male and female knockout mice as compared with controls and LH pulse profiles reported in the literature (23–25). These data are consistent with the observed higher GnRH levels and the possible increase in its release to the pituitary to stimulate LH release in these mutant mice.

The increase in LH levels impinged on the function of the gonads and the sexual behavior of *Nes-Cre:Selenot^fl/fl^* mice. In males, we found high circulating levels of T, but also E_2_, a condition that could be ascribed to a central effect exacerbating testicular steroidogenesis through increased LH levels. In contrast, circulating levels of FSH were lower in SELENOT-deficient mice, which could explain the testicular atrophy observed in these animals. In addition, the effects of these hormonal changes corroborate the reproductive dysfunction of the mutant animals, as observed when males with central SELENOT deficiency were mated with control females. These animals had severely reduced litter number and size, showing that the hormonal perturbations observed lead to marked hypofertility in knockout mice. This reproductive failure was also associated with altered mating behavior and a decrease in sexual maturation in males. Indeed, when placed with receptive control females, these males were unable to initiate mating, with longer latencies for mounting and intromission as compared with control mice. This behavior could be ascribed to altered T levels, which affect male sexual motivation and performance (26). This steroid hormone could also be converted to estrogens by a local aromatase to act through estrogen receptors (27, 28). These sex hormones are known to affect kisspeptin neuron activity in the ARC, where steroid hormone receptors are present (29). This neuroendocrine circuit is most likely altered in *Selenot*-knockout mice, as also supported by the reduced kisspeptin gene expression in the AVPV of *Nes-Cre:Selenot^fl/fl^* mice, which is known to control sexual behavior (30).

In SELENOT-deficient females, LH increase during puberty could lead to changes in sex hormone production and dynamic changes in ovaries. Indeed, these females exhibited low levels of FSH and E_2_, but high circulating levels of T. These findings indicate that exacerbated stimulation of the ovarian tissue by increased LH leads to increased production of T. This increase could also be due to an altered conversion of androgens to E_2_ by the aromatase in the thecal tissue (31). Conversely, FSH levels were decreased in brain-specific *Selenot*-knockout mice, which was associated with a decrease in the number of mature follicles producing E_2_. The origin of this decrease may arise from the altered production of FSH, which is known to control follicular development and estrogen production via T aromatization (32, 33). As a consequence of ovarian hormone changes and the global perturbation of the gonadotrope axis, ovarian cyclicity was deregulated, since *Selenot*-mutant mice spent more time in diestrus and less in proestrus compared with control littermates.

The fertility of female SELENOT-deficient mice was also investigated over 5 months, by mating with control males. These female mice displayed a very low gestation rate and gave birth to a low number of litters, which were smaller in size compared with control females. This hypofertility phenotype could be accounted for by the altered hormonal status observed in these mice. As in males, sex hormones strongly regulate female sexual behavior. Indeed, E_2_ level is the most critical factor regulating female sexual behavior (34, 35). The so-called lordosis reflex of receptive females, which is stimulated by mounting of a male, is regulated by E_2_ (36, 37). Indeed, it has been shown that local infusion of estrogens in the ventromedian nucleus of the hypothalamus, the mPOA, and the periaqueductal gray, areas with abundant sex hormone receptors, increases the lordosis reflex (38), indicating that estrogen release from the ovary directly affects the brain and promotes receptive behaviors. The low levels of E_2_ and decreased kisspeptin expression in the AVPV observed in female SELENOT-deficient mice are probably at the origin of the altered sexual behavior and hypofertility observed in these mice.

Central SELENOT deficiency also led to anatomical modifications of the gonads. In males, histological analyses revealed a testicular atrophy and pycnotic as well as vacuolar cells in seminiferous tubules. This could be ascribed to altered production of FSH, which is known to regulate testis development (39). In females, histological examination of ovaries revealed a depletion of antral follicles and corpora lutea, which is probably related to LH excess (40, 41). We also found cysts in the ovary of female *Nes-Cre:Selenot^fl/fl^* mice, which recall PCOS. This frequent cause of infertility in women is characterized by higher androgen levels and oligoovulation, in addition to the presence of cysts in the ovaries, features also found in brain SELENOT-deficient mice. It is now well established that the central neuroendocrine reproductive system is affected in this pathology (42). Indeed, the typical cyclical changes in frequency of GnRH release are often absent in PCOS, resulting in a persistent high-frequency drive inducing gonadotropin changes (i.e., high LH and low FSH levels) that are responsible of hyperandrogenemia and ovulatory dysfunction (43, 44). These changes are reminiscent of the *Selenot*-knockout phenotype described in the present study. It is interesting to note that the hyperglycemia observed in a proportion of patients with PCOS (45) was also found in female *Nes-Cre:Selenot^fl/fl^* mice, a change which could be ascribed to the FSH decrease observed in these mice, as this hormone has been shown to contribute to glucose-induced insulin release from pancreatic β cells (46). Since the specific mechanisms underlying the GnRH neuron dysfunction in PCOS remain unclear, changes in SELENOT gene expression in this pathology and the potentially associated ER and oxidative stress, known to be regulated by this thioredoxin-like enzyme, should be further considered and investigated as a possible etiological element of the disease. Of note, serum selenium and selenoprotein P are inversely associated with oxidative stress biomarkers and positively associated with total antioxidant capacity in patients with PCOS, suggesting that selenoproteins could be involved in the etiology of the disease (47).

In order to confirm the involvement of GnRH overproduction in the phenotype of *Selenot* conditional–knockout mice, we sought to reverse the hormonal changes observed by administering a GnRH antagonist. Interestingly, intermittent administration of the GnRH antagonist cetrorelix at a concentration that competes with endogenous GnRH (18) was able to restore the estrous cyclicity and to promote the pulsatile release of LH at levels comparable to those of controls in both males and females. These data demonstrate that GnRH overproduction is responsible for the neuroendocrine and reproductive disturbances observed in SELENOT-deficient mice.

Increased GnRH production and gene expression evidenced in the present study indicate that SELENOT could be involved in the synthesis and secretion of GnRH, likely through redox balance regulation in GnRH neurons expressing SELENOT. As an ER antioxidant protein, SELENOT could ensure appropriate GnRH production and release through the regulation of key redox proteins whose impairment could lead to dysregulated release of the neurohormone. This may in turn trigger a feed-forward mechanism at the gene expression level to sustain high intracellular concentrations of GnRH. In support of this hypothesis, treatment of mutant mice with cetrorelix acetate, which competes with GnRH for binding to GnRH receptors, thus decreasing or blocking GnRH action, reverted the neuroendocrine changes observed in these mice. We are currently exploring this possibility by specifically knocking down SELENOT in GnRH neurons.

SELENOT deficiency could also affect other neuronal populations, as previously shown for catecholaminergic neurons (12, 13), including afferent neurons that regulate GnRH output. It is well known that different neurotransmitters and neuropeptides regulate GnRH neuron activity (48). These include glutamate, GABA, dopamine, NPY, and kisspeptin, among others, which play a critical role in pubertal activation of GnRH neurons in mammals (6, 7, 49). SELENOT might play a role in the availability of some of these regulatory cues. It is now well known that KNDy neurons’ signaling through kisspeptin, neurokinin B, and dynorphin release from the ARC is the GnRH pulse generator (23). We found in the present study that kisspeptin gene expression is altered in the AVPV but not in the ARC, indicating that other regulatory cues or mechanisms could be responsible for LH pulse changes observed in *Nes-Cre:Selenot^fl/fl^* mice. Indeed, it has been shown that glutamate signaling, for example during diestrus, inhibits LH pulses (24). In addition, gonadal steroid levels, which are affected in *Nes-Cre:Selenot^fl/fl^* mice, also modulate pulsatile GnRH dynamics (50). Alternatively, the mode of release of kisspeptin and not its gene expression could be altered following *Selenot* gene disruption, leading to changes in LH pulse dynamics. Further studies will be required to better understand the molecular mechanisms leading to the neuroendocrine phenotype observed in *Nes-Cre:Selenot^fl/fl^* mice and their potential developmental origin.

Of note, we recently showed that dopaminergic pathways are altered in the hypothalamic area of both male and female SELENOT-deficient mice (15). Since dopamine, acting through D1 and D2 receptors, can inhibit the activity of GnRH neurons in the AVPV (51), it is conceivable that the effects of SELENOT inactivation on the dopaminergic circuitry could also result in defects in the gonadotrope axis.

In conclusion, the data obtained in the present study uncover a previously unknown role of a selenoprotein, i.e., SELENOT, in the central control of reproduction in mammals, thus offering an opportunity to unveil unexplored potential pathological mechanisms underpinning fertility disorders.

## Methods

### Sex as a biological variable.

Sex was considered as a biological variable, and both male and female mice were used in the present study.

### Animals.

Mice were housed under a 12-hour light/dark cycle (light on at 07:00 am) and had access to food and water ad libitum. Ambient temperature was maintained at 22°C ± 2°C. To generate transgenic mice, the *Selenot* gene was floxed by homologous recombination in the genome of C57BL/6J mice to delete the sequence region encoding the thioredoxin-like domain (exons 2 and 3). Mice with *Selenot^fl/fl^* allele were generated in the Mouse Clinical Institute (IGBMC, INSERM, CNRS, Strasbourg University, France) where they are available. They were then bred with Cre recombinase–expressing mice to promote the genetic deletion of the thioredoxin-like motive. The brain conditional mutant *Nes-Cre:Selenot^fl/fl^* mouse (12, 13) was generated by mating *Selenot^fl/fl^* mice with animals expressing Cre recombinase under the control of the rat nestin (*Nes*) promoter (*Tg* [*Nestin-Cre*]*1 Kln^+/–^* knockin mice), which were acquired from The Jackson Laboratory (12). The genotypes were determined by PCR with the following primers: forward, 5′GGCTTTATGTAAGCAGTTCTAAACTGTTTCTGC-3′, and reverse, 5′-CGCCCCATTTTATAAACTTTGTATGTTTATGCCC-3′, for control (211 bp) and *Selenot^fl^* (261 bp) alleles; forward, 5′-ATCGCCAGGCGTTTTCTGAGCATAC-3′, and reverse, 5′-GCCAGATTACGTATATCCTGGCAGC-3′, for the *Cre* allele (387 bp); and forward, 5′-GGCTTTATGTAAGCAGTTCTAAACTGTTTCTGC-3′, and reverse, 5′-GCCTAGGTTTTACCTGAGAAACCAAAGG-3′, for the *Selenot^–^* allele (409 bp). Littermate *Nes*-Cre mice obtained after intercrossing heterozygous mice carrying WT, floxed, and *Nes*-Cre alleles were used as controls. No significant difference in body weight or fat mass between *Nes-Cre:Selenot^fl/fl^* and control mice was observed.

### Assessment of estrous cyclicity and fertility.

The estrous cycle stage was assessed through cytological analysis of vaginal smears of adult mice (52). The reproductive competency of the animals was determined by mating animals for a period of 5 months. Fertility index (number of litters per female over 5 months) and number of pups/litter (litter size) were determined, and pregnancy rate was calculated as the ratio of the number of pregnant females to the number of females with successful mating.

### Sexual behavior.

Three-month-old, sex-experienced male and female mice were individually housed for 1 week before being used. To evaluate sexual behavior, each male was placed into a cage for 10 minutes for adaptation before a female was introduced into the cage. Female mice that were in natural estrus phase were used. The test lasted 2 hours and was conducted under red light illumination during the dark phase of the light/dark cycle. It was videotaped and scored manually as previously described (53). The following targeted measures were recorded and scored: mount latency (defined as the time between introduction of the female mice and the first mount), intromission latency (considered as the time between introduction of the female mice and the first intromission), mount frequency (which is the number of mounts), intromission frequency (which is the number of intromissions), and copulatory efficacy (which is calculated as intromission frequency divided by mount frequency + intromission frequency) (54). Mount was defined as a male using both fore paws to climb onto a female from behind for copulation. Intromission was defined as a male pelvic thrust with a stable frequency continuously, and demonstration of the female anogenital area elevated over the ground when finished. The lordosis response was defined as a female with all 4 paws grounded, with the hind region elevated from the floor of the test chamber, and no evidence of attempt to escape or exhibition of a defensive upright posture, and the back slightly arched (54). A lordosis quotient was calculated by dividing the number of lordosis responses displayed by the female mice by the number of mounts received (55).

### Tissue perfusion and immunohistochemistry.

Adult mice were anesthetized using a solution of ketamine (100 mg/kg) and xylazine (10 mg/kg) and then perfused transcardially with 0.9% NaCl in 0.1 M phosphate buffer (pH 7.4), followed by 4% paraformaldehyde (PFA) in phosphate-buffered saline (PBS). Brains and gonads were removed and postfixed in the same fixative at 4°C, which was changed to PBS/azide after 24 hours. Tissues were sectioned into 40-μm slices with a vibratome. The sections were incubated with 1% donkey serum diluted in 1% bovine serum albumin and 0.3% Triton X-100 in PBS for 2 hours at room temperature, and then exposed overnight at 4°C to primary antibodies, including rabbit anti-SELENOT diluted 1:200 (11) and anti-GnRH diluted 1:5000 (56, 57). As controls, incubation with the peptides used to generate the antibodies abolished the immunoreactions. Immunostaining was visualized using Alexa Fluor 488– or 594–conjugated secondary antibodies diluted 1:200 (Invitrogen). Counterstaining with 1 μg/mL 4,6-diamino-2-phenylindole (DAPI; Sigma-Aldrich) in PBS for 1 minute was performed before mounting the slides with PBS/glycerol (50:50). Samples were analyzed with a Leica SP8 confocal laser-scanning microscope (DMRAX-UV, Leica Microsystems).

### LH, FSH, T, and E_2_ levels.

LH levels were determined by a sandwich ELISA, as described previously (58). Briefly, a 96-well high-affinity binding microplate (Corning) was coated with a bovine LHβ518B7 monoclonal antibody (1:1000 in PBS; 50 μL per well), and circulating hormone levels were determined using a mouse LH-RP reference provided by Albert F. Parlow (National Hormone and Peptide Program, Harbor-UCLA Medical Center, Torrance, California, USA), a rabbit polyclonal primary antibody for LH (1:10,000; rabbit antiserum, AFP240580Rb; National Hormone and Peptide Program), and a polyclonal goat anti-rabbit IgG secondary antibody (1:1000; DAKO). LH pulses were determined on blood samples, acquired every 10 minutes over a period of 2 hours between 10:00 am and 12:00 pm. Commercially available mouse FSH (AssayGenie,), T (Demeditec Diagnostics, GmnH), and E_2_ ELISA (Cayman Chemical) kits were used to measure plasma FSH, T, and E_2_, respectively. Control levels were in accordance with those described in the manufacturer’s instructions.

### Ovarian histology.

Ovaries were collected from adult females at diestrus, immersion-fixed in 4% PFA solution, and stored at 4°C. Paraffin-embedded ovaries were sectioned at a thickness of 5 μm (histology facility, University of Lille 2) and stained with H&E (Sigma-Aldrich). Sections were visualized with a light microscope (Zeiss Axioscope) and examined throughout the ovary. Antral follicles and corpora lutea were identified and quantified as previously reported (59).

### Testis histology.

Testicular tissue was fixed for 12 hours in Bouin’s fixative (Sigma-Aldrich) at room temperature, then dehydrated in graded baths of ethanol and embedded in paraffin. Sections (3 μm thick) were cut using a microtome (Leica Microsystems GmbH). Serial tissue sections were mounted on each Polysine slide (Thermo Fisher Scientific). Two slides (no. 1 and 5) were examined to obtain an accurate and global assessment of the tissue. Slides were coded for blinded analysis and stained with H&E and saffron to obtain an accurate appreciation of seminiferous tubule architecture, as previously described (60). Serial digital images were obtained with a light microscope (Leica Microsystem GmbH) equipped with Leica Application Suite software (Leica Microsystem GmbH). The ratio of the number of pyknotic cell nuclei to the total surface area of each seminiferous tubule was evaluated for a total of 100 seminiferous tubules per testis. The number of pyknotic cells per seminiferous tubule presenting at least 1 pyknotic cell nucleus (number of pyknotic cell/1000 μm^2^) was assessed. In addition, the percentage of seminiferous tubules with vacuolization was also evaluated.

### iDISCO: whole-brain immunostaining.

Mice were anesthetized through i.p. injection of a mixture of ketamine (100 mg/kg body weight) and xylazine (10 mg/kg body weight), and perfused with PBS followed by 4% PFA. Brains were extracted and postfixed in 4% PFA overnight at 4°C. Fixed brains were washed in PBS for 1 hour twice, then in 20%, 40%, 60%, and 80% methanol (in H_2_O) and 100% methanol twice for 1 hour. They were then bleached with 5% H_2_O_2_ in 100% methanol overnight at 4°C in the dark without shaking. Brains were rehydrated successively in 80%, 60%, 40%, and 20% methanol, and PBS, each step lasting 1 hour before staining procedures. Pretreated brains were incubated in a permeabilization solution containing 0.2% Triton X-100, 20% DMSO, and 0.3 M glycine in PBS at 37°C for 2 days with shaking. Then, the brains were transferred to a blocking solution (0.2% Triton X-100, 6% donkey serum, and 10% DMSO in PBS) and incubated at 37°C for 2 days with shaking. The brains were then washed in a solution of PBS containing 0.2% Tween 20 and 10 μg/mL heparin for 36 hours, and incubated with the rabbit anti-GnRH primary antibody diluted 1:5000 in the incubation solution containing 0.2% Tween 20, 5% DMSO, 10 μg/mL heparin, and 3% donkey serum in PBS, for 7 days at 37°C with shaking. The brains were then washed with the washing solution described above for 1 day, and then incubated with the donkey anti-rabbit Alexa Fluor secondary antibody (Interchim) diluted 1:500 in the incubation solution containing 0.2% Tween 20, 10 μg/mL heparin, and 3% donkey serum in PBS, for 7 days at 37°C with shaking. The brains were then washed in the washing solution for 1 day.

### Clearing procedure.

First, the brains were dehydrated in methanol/H_2_O solution (20%, 40%, 60%, and 80%, 1 hour each) and 100% methanol for 1 hour twice with shaking. The brains were then incubated overnight in 1 volume of 100% methanol and 2 volumes of 100% anhydrous dichloromethane (Sigma-Aldrich) at room temperature, and washed twice for 15 minutes each in 100% dichloromethane with shaking. Finally, the brains were incubated in 100% dibenzyl ether (Sigma-Aldrich) for at least 3 hours with shaking before imaging.

### Light-sheet imaging.

Cleared samples were imaged with an Ultramicroscope II (LaVision BioTec) using the ImspectorPro software (LaVision BioTec). The light sheet was generated by a laser at wavelengths of 561 nm or 640 nm (Coherent Sapphire Laser, LaVision BioTec) and 6 cylindrical lenses. A binocular stereomicroscope (MXV10, Olympus) with a 2× objective (MVPLAPO, Olympus) was used at ×63 magnification. 3D images were constructed from a *Z*-series of Ultramicroscope II fluorescence images using Imaris software, version 9.0.0_64 (http://bitplane.com). 3D pictures and tiff series were obtained using the “Snapshot” and the “animation” tools of the Imaris software. Semiautomatic counting of GnRH neurons was done with the “spot detection” tool of the Imaris software, and a manual verification was used with the “clipping plan” tool.

### GnRH antagonist treatment.

LH pulse frequency in adult male and female control and *Nes-Cre:Selenot^fl/fl^* mice was analyzed after injection of the GnRH antagonist cetrorelix acetate (Sigma-Aldrich). For this, male and female mice were treated with i.p. injections of 200 μL of 0.01 M PBS (pH 7.4) or a solution containing 0.5 mg/kg/day of cetrorelix acetate in 0.01 M PBS (pH 7.4) for 3 days (18). After treatment, serial blood sampling from males and diestrous females was performed for LH pulse frequency assessment. Estrous cyclicity and LH levels were also analyzed before, during, and after treatment with cetrorelix acetate. For this, adult females were cycled for 12 days before the onset of treatment. Vaginal cytology was analyzed to record the specific days of the estrous cycle. Females were injected i.p. with 200 μL of a solution containing 0.5 mg/kg of cetrorelix acetate in 0.01 M PBS (pH 7.4), every second day during the treatment period. Tail-blood samples were collected for LH measurements 1 time before the beginning of the treatment and on days 2 and 6 during treatment, as well as day 4 after the last injection. Estrous cyclicity was monitored daily between 8 and 10 am, at the period before, during, and after treatment with the GnRH antagonist.

### Quantitative PCR analysis.

Total RNA was extracted using the Nucleospin RNA kit (Macherey-Nagel). One microgram of RNA was reverse transcribed with the ImProm-II Reverse Transcription system (Promega) according to the supplier’s recommendations. cDNA amplification was carried out using the SYBR Green PCR Mastermix (Applied Biosystems). Quantitative PCR was carried out in the QuantStudio Flex system (Applied Biosystems), and gene expression was normalized to the housekeeping genes GAPDH and β-actin by the 2^–ΔΔCt^ method. The primers used were mouse GnRH forward, 5′-GCATTCTACTGCTGACTGTGTGTT-3′, and reverse, 5′-GTTCTGCCATTTGATCCACCT-3′; mouse GAPDH forward, 5′-AGTGTTTCCTCGTCCCGTAGAC-3′, and reverse, 5′-CGCCCAATACGGCCAAA-3′; mouse β-actin forward, 5′-AGATGACCCAGATCATGTTTGAGA-3′, and reverse, 5′-CACAGCCTGGATGGCTACGT-3′.

### RNAScope fluorescence in situ hybridization.

In situ mRNA hybridization was performed using the RNAscope Multiplex Fluorescent Reagent Kit v2 (ACD Biotechne). Briefly, mice were anesthetized and perfused with 4% PFA. The dissected brains were postfixed with 4% PFA overnight and dehydrated with 30% sucrose in PBS. Then, the brains were cryosectioned into 12-μm sections and used for in situ hybridization. All the procedures were carried out according to the manufacturer’s protocol and as described previously (15). SELENOT (NM_001040396.3), GnRH (XM_006518564.3), and kisspeptin (XM_006529679.2) probes were supplied by ACD Biotechne. Staining of the nuclei was done with RNAscope DAPI (ACD Biotechne), and the slides were mounted with Fluoromount (Sigma-Aldrich). The hybridization signals were acquired with a Leica SP8 confocal laser-scanning microscope (DMRAX-UV, Leica Microsystems) and analyzed using ImageJ/Fiji software, and relative mean fluorescence intensity for each condition was calculated.

### Statistics.

In the experiments with mice, matched controls from the same litters were used. Results are expressed as mean ± SEM. Statistical analysis of the data was performed with Prism 9 software (GraphPad). Statistical comparisons between groups were made using a 2-tailed, unpaired Student’s *t* test or the nonparametric Kruskal-Wallis test with a post hoc Dunn’s test.

### Study approval.

All animal experimental procedures were reviewed by the Normandy Ethics Committee on Animal Experimentation, and approved by the French Higher Education and Research Ministry under authorization no. 43707-2023051712103580 carried out in compliance with the European Committee Council directives.

### Data availability.

Values for all data points in graphs are reported in the Supporting Data Values file.

## Author contributions

BYM conducted experiments, analyzed results, and wrote the manuscript. LB, LD, AA, MG, AP, DA, and DG performed experiments and analyzed results. YT and LG provided tools and analyzed results. NR and FC analyzed results. YA conceived and designed experiments, analyzed results and wrote the manuscript. All authors have reviewed and approved the manuscript.

## Funding support

- INSERM.
- University of Rouen Normandy.
- Regional Council of Normandy.
- National Agency of Research (ANR).

## Supplementary Material

Supporting data values

## Version 1 11/06/2025

In-Press Preview

## Version 2 12/22/2025

Electronic publication
